# Computational Analysis of the Model Describing HIV Infection of CD4^+^T Cells

**DOI:** 10.1155/2014/618404

**Published:** 2014-07-17

**Authors:** Abdon Atangana, Emile Franc Doungmo Goufo

**Affiliations:** ^1^Institute for Groundwater Studies, Faculty of Natural and Agricultural Sciences, University of the Free State, Bloemfontein 9300, South Africa; ^2^Department of Mathematical Sciences, University of South Africa, Florida Sciences Campus, Florida 0003, South Africa

## Abstract

An analysis of the model underpinning the description of the spread of HIV infection of CD4^+^T cells is examined in detail in this work. Investigations of the disease free and endemic equilibrium are done using the method of Jacobian matrix. An iteration technique, namely, the homotopy decomposition method (HDM), is implemented to give an approximate solution of nonlinear ordinary differential equation systems. The technique is described and illustrated with numerical examples. The approximated solution obtained via HDM is compared with those obtained via other methods to prove the trustworthiness of HDM. Moreover, the lessening and simplicity in calculations furnish HDM with a broader applicability.

## 1. Introduction

The cause of the progressive depletion of CD4^+^T cells in HIV-infected people is one of the most fundamental and controversial issues in AIDS research. HIV infects and kills CD4^+^T cells. The infection results in high T-cell activation and turnover. An immediately intuitive assumption is that HIV-mediated destruction of CD4^+^ cells directly reduces the number of these cells and that the high turnover rates of T cells and the slow progression to AIDS reflect a long but eventually lost struggle of the immune system to replace killed cells in its effort to maintain T-cell homeostasis [1–4]. However, HIV mainly infects activated CD4^+^ cells, and activated cells normally follow different dynamics than cells that belong to resting populations whose numbers are controlled by homeostatic mechanisms.

In this study, we consider that the HIV infection model of CD4^+^T cells is examined in [5]. This model is given by the components of the basic three-component model which are the concentration of susceptible CD4^+^T cells, CD4^+^T cells infected by the HIV viruses, and free HIV virus particles in the blood. CD4^+^T cells are also called leukocytes or T helper cells. These with order cells in human immunity systems fight against diseases. HIV use cells in order to propagate. In a healthy person, the number of CD4^+^T cells is 800/1200 mm^3^. This model is characterized by a system of the nonlinear differential equations:

(1) dTdt=p−αT+rT(1−T+ITmax⁡)−kVT,dIdt=kVT−βI,dVdt=NβI−γV,

subject to the initial conditions

(2) $$\begin{array}{c} T\left(0\right)=T_{0},\;\;I\left(0\right)=I_{0},\;\;V\left(0\right)=V_{0}. \end{array}$$

Here, *r* is any positive constant, *T*(*t*), *I*(*t*), and *V*(*t*) show the concentration of susceptible CD4^+^T cells, CD4^+^T cells infected by the HIV viruses, and free HIV virus particles in the blood, respectively, *α*, *β*, and *γ* stand for natural turnover rates of uninfected Tells, infected T cells, and virus particles, respectively, (1 − (*T* + *I*)/*T*_max⁡_) describes the logistic growth of the healthy CD4^+^T cells, and proliferation of infected CD4^+^T cells is neglected. For *k* > 0 is the infection rate, the term *KVT* describes the incidence of HIV infection of healthy CD4^+^T cells. Each infected CD4^+^T cell is assumed to produce l virus particles during its lifetime, including any of its daughter cells. The body is believed to produce CD4^+^T cells from precursors in the bone marrow and thymus at a constant rate *p*. T cells multiply through mitosis with a rate *r* when T cells are stimulated by antigen or mitogen. *T*_max⁡_ denotes the maximum CD4^+^T cell concentration in the body [6–9].

As Andrianov and Manevitch wrote in the foreword of a popular science book* Asymtotology: Ideas, Methods, and Applications*, asymptotic methods belong to the, perhaps, most romantic area of modern mathematics [10]. Though computer science is growing very fast and numerical simulation is applied everywhere, nonnumerical issues will still play a large role [11–14]. There exist some alternative analytical asymptotic approaches, such as the nonperturbative method, modified Lindstedt-Poincaré method [13], variational iteration method [14], Adomian decomposition method [15], homotopy perturbation method [16, 17], and book-keeping artificial parameter perturbation method [18].

The purpose of this paper is to derive analytical solutions of model for HIV infection of CD4^+^T cells (1) via the relatively new analytical solution homotopy decomposition method (HDM). The HDM was recently used to solve one- and two-dimensional fractional heat-like problem, Burgers equation, the Klein-Gordon equation, a coupled Burgers equation, 2D and 3D Poisson equations and biharmonic equations, the groundwater flow equation [19], the Hamilton-Jacobi-Bellman equation, high even-order differential equation, parabolic equations and coupled Van der Pol's nonlinear partial differential equation, and coupled attractor one-dimensional Keller-Segel equations. This method displays some advantages over the existing methods.

## 2. Stability Analysis

The aim of this section is to present a stability analysis of the system equation (1) that will be achieved via the use the eigenvalues obtained via the Jacobian matrix. We will start by providing the equilibrium point and also the desease free equilibrium. To obtain the equilibrium points, we assume that the system does not depend on the parameter *t*; this will further imply that

(3) 0=p−αT−+rT−(1−T−+ITmax⁡)−kV− T−,0=kV− T−−βI  −,0=NβI−−γV−.

After the first manipulations, we obtain that

(4) $$\begin{array}{c} \overset{-}{I}=\frac{k\overset{-}{V}\;\overset{-}{T}}{\beta }=\frac{\gamma \overset{-}{V}}{N\beta }. \end{array}$$

From the above equation, we can obtain

(5) $$\begin{array}{c} \overset{-}{T}=\frac{\gamma }{N\beta }. \end{array}$$

However, replacing (5) into the first equation of system (3) and rearranging it, we obtain the following:

(6) I−=p+((rγ−γ2)/Nβ)−(rγ2/(Nβ2)Tmax⁡)(rγ/NβTmax⁡)+β,V−=Nβγp+((rγ−γ2)/Nβ)−(rγ2/(Nβ2)Tmax⁡)(rγ/NβTmax⁡)+β.

Therefore, the equilibrium points are given as

(7) (I−,V−,T−)=(p+((rγ−γ2)/Nβ)−(rγ2/(Nβ2)Tmax⁡)(rγ/NβTmax⁡)+β,      Nβγp+((rγ−γ2)/Nβ)−(rγ2/(Nβ2)Tmax⁡)(rγ/NβTmax⁡)+β,    γNβ).

The above equilibrium points are valid if the following conditions are satisfied:

(8) $$\begin{array}{c} p+\frac{r\gamma -\gamma^{2}}{N\beta }-\frac{r\gamma^{2}}{{\left(N\beta \right)}^{2}T_{\max}}\geq 0. \end{array}$$

The disease free equilibrium is obtained by solving the following equation:

(9) $$\begin{array}{c} -\frac{r}{T_{\max}}{\overset{-}{T}}^{2}+\left(r-\alpha \right)\overset{-}{T}+p=0, \end{array}$$

which has the following solution:

(10) $$\begin{array}{c} \overset{-}{T}=\frac{r-\alpha \mp \sqrt{{\left(r-\alpha \right)}^{2}+\left(4r/T_{\max}\right)}}{2\left(r/T_{\max}\right)}. \end{array}$$

Therefore, the diseases free equilibrium is given as

(11) $$\begin{array}{c} \left(\overset{-}{I},\overset{-}{V},\overset{-}{T}\right)=\left(0,0,\frac{r-\alpha +\sqrt{{\left(r-\alpha \right)}^{2}+\left(4r/T_{\max}\right)}}{2\left(r/T_{\max}\right)}\right). \end{array}$$

With the above information on hand, we will now find the eigenvalues associated with this problem, which will allow us to give stability of the system. The Jacobian matrix associated with this problem is given by

(12) $$\begin{array}{c} J=\left(\begin{array}{ccc} r-\alpha -kV-\frac{2rT}{T_{\max}} & -\frac{T}{T_{\max}} & -kT\\ kV & -\beta & kT\\ 0 & N\beta & -\gamma \end{array}\right). \end{array}$$

Now, using the equilibrium free point, we obtain the following:

(13) $$\begin{array}{c} J=\left(\begin{array}{ccc} r-\alpha -\frac{2rT_{\text{free}}}{T_{\max}} & -\frac{T_{\text{free}}}{T_{\max}} & -kT\\ 0 & -\beta & kT_{\text{free}}\\ 0 & N\beta & -\gamma \end{array}\right). \end{array}$$

In order to find the eigenvalues associate, we will solve the following equation:

(14) $$\begin{array}{c} \det\left(J-\lambda I\right)=\left(\begin{array}{ccc} r-\alpha -\frac{2rT_{\text{free}}}{T_{\max}}-\lambda & -\frac{T_{\text{free}}}{T_{\max}} & -kT\\ kV & -\beta -\lambda & kT_{\text{free}}\\ 0 & N\beta & -\gamma -\lambda \end{array}\right)=0. \end{array}$$

Using the standard method, we obtain the following solution:

(15) λ1=r−α−2rTTmax⁡,λ2=−β−γ−(β−γ)2+4NβTfree2,λ3=−β−γ+(β−γ)2+4NβTfree2.

With the Eigen values in hand, we conclude that there is stability if and only if the following conditions are observed:

(16) $$\begin{array}{c} r-\alpha -\frac{2rT}{T_{\max}}<0,\;\;-\beta -\gamma +\sqrt{{\left(\beta -\gamma \right)}^{2}+4N\beta T_{\text{free}}}<0. \end{array}$$

## 3. Some Useful Information regarding the Methodology of (HDM)

To illustrate the basic idea of this method, we consider a general nonlinear nonhomogeneous differential equation with initial conditions of the following form (see also in [16, 19]):

(17) $$\begin{array}{c} \frac{\partial^{m}U\left(t\right)}{\partial t^{m}}=L\left(U\left(t\right)\right)+N\left(U\left(t\right)\right)+f\left(t\right),\;m=1,2,3,\ldots . \end{array}$$

Subjected to the initial condition,

(18) $$\begin{array}{c} \frac{\partial^{i}U\left(0\right)}{\partial t^{i}}=y_{i},\;i=0,1,2,\ldots ,m-1. \end{array}$$

*m* is the order of the derivative.

*f* is a known function, *N* is the general nonlinear differential operator, *L* represents a linear differential operator, and *m* is the order of the derivative. The method's first step here is to apply the inverse operator ∂^*m*^/∂*t* ^*m*^ on both sides of (17) to obtain

(19) U(t)=∑k=0m−1tkk!yk  +∫0t∫0t1⋯∫0tm−1L(U(τ))+N(U(τ))          +f(τ)dτ⋯dt.

The multi-integral in (17) can be transformed to

(20) ∫0t∫0t1⋯∫0tm−1L(U(τ))+N(U(τ))+f(τ)dτ⋯dt =1(m−1)!∫0t(t−τ)m−1L(U(τ))+N(U(τ))+f(τ)dτ

so that (19) can be reformulated as

(21) U(t)=∑k=0m−1tkk!yk+1(m−1)!  ×∫0t(t−τ)m−1L(U(τ))    +N(U(τ))+f(τ)dτ.

Using the homotopy scheme, the solution of the above integral equation is given in series form as follows:

(22) $$\begin{array}{c} U\left(t,p\right)=\overset{\infty }{\underset{n=0}{\sum }}p^{n}U_{n}\left(t\right),\\ U\left(t\right)=\underset{p\to 1}{\lim}U\left(t,p\right), \end{array}$$

and the nonlinear term can be decomposed as

(23) $$\begin{array}{c} NU\left(t\right)=\overset{\infty }{\underset{n=1}{\sum }}p^{n}H_{n}\left(U\right), \end{array}$$

where *p* ∈ (0, 1] is an embedding parameter. *H*_*n*_(*U*) is the He's polynomials that can be generated by

(24) Hn(U0,…,Un)=1n!∂n∂pn[N(∑j=0npjUj(t))],n=0,1,2,….

The homotopy decomposition method is obtained by the graceful coupling of decomposition method with He's polynomials and is given by

(25) ∑n=0∞pnUn(t)=T(t)+p1(m−1)!       ×∫0t(t−τ)m−1[f(τ)+L(∑n=0∞pnUn(τ))                 +∑n=0∞pnHn(U)]dτ,

with

(26) $$\begin{array}{c} T\left(t\right)=\overset{m-1}{\underset{k=0}{\sum }}\frac{t^{k}}{k!}y_{k}. \end{array}$$

Comparing the terms of the same powers of *p*, we obtain solutions of various orders. The initial guess of the approximation is *T*(*t*); this is actually the Taylor series of the exact solution of order *m*. Note that this initial guess insures the uniqueness of the series decompositions.

## 4. Applications

In this section, we applied the HDM to solve the system of the nonlinear differential equations (1). To be consistent in comparison with the existing methods, we chose the following initial condition and parameters as in [20, 21]:

(27) $$\begin{array}{c} T_{0}=0.1,\;\;I_{0}=0,\;\;V_{0}=0.1,\;\;p=0.1,\\ \alpha =0.02,\;\;\beta =0.3,\;\;\gamma =0.3,\\ k=0.0027,\;\;\;T_{\max}=1500,\;\;N=10. \end{array}$$

Following carefully the steps of the HDM, we obtain the following equations:

(28) ∑n=0∞pnTn(t)=T(0)+p∫0t(p−α∑n=0∞pnTn(τ)+r∑n=0∞pnTn(τ)       ×(1−∑n=0∞pnTn(τ)+∑n=0∞pnIn(τ)Tmax⁡)         −k∑n=0∞pnTn(τ)∑n=0∞pnVn(τ))dτ,∑n=0∞pnIn(t)=  I(0)+p∫0t(k∑n=0∞pnTn(τ)∑n=0∞pnVn(τ)                 −β∑n=0∞pnIn(τ))dτ,∑n=0∞pnVn(t)=V(0)+p∫0t(βN∑n=0∞pnTn(τ)                 −γ∑n=0∞pnVn(τ))dτ.

Comparing the terms of the same power of *p*, we obtain the following integral equations that are much easier to solve. Note that, with the homotopy perturbation method (HPM), one will obtain a set of ordinary differential equations, after comparing the terms of the same power of *p*, which something is very heavy to compute in the case of high order ODE. Consider

(29) p0=T0(t)=T0,  T0(0)=T0,p0=I0(t)=I0,    I0(0)=I0,p0=V0(t)=V0,    V0(0)=V0,p1: T1(t)∫0t(p−αT0+rT0(1−T0+I0Tmax⁡)−kV0T0)dτ,   T1(0)=0p1: I1(t)=∫0t(kV0T0−βI0)dτ,  I1(0)=0p1: V1(t)=∫0t(NI0−γV0)dτ,  V1(0)=0    ⋮pn: Tn(t)=∫0t((r−α)Tn−1            −rTmax⁡(∑j=0n−1TjTn−j−1+∑j=0n−1TjIn−j−1)          −k∑j=0n−1TjVn−j−1)dτ,  Tn(t)=0, n≥2pn: In(t)=∫0t(k∑j=0n−1VjTn−j−1−βIn−1)dτ,  In(t)=0,   n≥2pn: Vn(t)=∫0t(NβIn−1−γVn−1)dτ,  Vn(t)=0,   n≥2.

We will give the general algorithm in order to accommodate scholars using computers program; the algorithm will then be used to derive special solution of the system of equation for a given set of theoretical parameters.

**Algorithm 1 .**

- input: *J*_1_(*t*), *J*_2_(*t*), and *J*_3_(*t*) as initial guest;
- *j*-number terms in the rough calculation;
- output: *T*_approx_(*t*), *I*_approx_(*t*),  and *V*_approx_(*t*) the approximate solutions.

*Step 1*. Put *T*_0_(*t*) = *T*_0_, *I*_0_(0) = *I*_0_, *V*_0_(*t*) = *V*_0_, *T*_approx_(*t*) = *T*_0_(*t*), *I*_approx_(*t*) = *I*_0_(*t*)   and   *V*_approx_(*t*) = *V*_0_(*t*).

*Step 2*. From *j* = 0 to *n* − 1, do Steps 3 and Step 4.

*Step 3*. Compute

(30) Tn(t)=∫0t((r−α)Tn−1−rTmax⁡(∑j=0n−1TjTn−j−1+∑j=0n−1TjIn−j−1)       −k∑j=0n−1TjVn−j−1)dτIn(t)=∫0t(k∑j=0n−1VjTn−j−1−βIn−1)dτ,Vn(t)=∫0t(NβIn−1−γVn−1)dτ.

*Step 4*. Compute *T*_approx_(*t*) = *T*_approx_(*t*) + *T*_*n*+1_(*t*), *I*_approx_(*t*) = *I*_approx_(*t*) + *I*_*n*+1_(*t*), and *V*_approx_(*t*) = *V*_approx_(*t*) + *V*_*n*+1_(*t*).

Stop.

We will now make use of the above algorithm to derive the special solution. We therefore obtain the following series solutions:

(31) T0(t)=T0=0.1,  I0(t)=I0=0,V0(t)=V0=0.1,T1(t)=0.397953t,  I1(t)=0.000027t,V1(t)=−0.24t,T2(t)=0.592849t2,  I2(t)=0.0000172737t2,V2(t)=0.288041t2,T3(t)=0.588719t3,  I3(t)=−8.40515×10−6t3,      V3(t)=−0.230415t3,T4(t)=0.43829519t4,  I4(t)=6.14727882×10−6t4,V4(t)=0.1382427719t4,T5(t)=0.2608633362t5,  I5(t)=2.835861862×10−6t5,V5(t)=−6.635284213×10−2t5,T6(t)=0.1291947662t6,  I6(t)=1.153299855×10−5t6,V4(t)=2.653971892t6,T7(t)=0.01363544556t7,I7(t)=−3.925609032×10−7t7,V7(t)=2.481235575×10−6t7,T8(t)=−2.787485189×10−5t8,I8(t)=4.5660570151×10−11t8,V8(t)=1.28523993×10−10t5.

Using the package Mathematica, in the same manner, one can obtain the rest of the components. But, in this case, 9 terms were computed and the asymptotic solution is given by

(32) T(t)=T0(t)+T1(t)+T2(t)+T3(t)+T4(t)   +T5(t)+T6(t)+T7(t)+T8(t)+⋯I(t)=I0(t)+I1(t)+I2(t)+I3(t)+I4(t)   +I5(t)+I6(t)+I7(t)+I8(t)+⋯V(t)=V0(t)+V1(t)+V2(t)+V3(t)+V4(t)   +V5(t)+V6(t)+V7(t)+V8(t)+⋯.

### 4.1. Numerical Applications

To test the effectiveness and the accuracy of the HDM for solving this type of problem, we compare the approximated solutions obtained via other methods and HDM, and the results are shown in Tables 1, 2, and 3.

Numerical comparison shows that the approximated solutions obtained via LADM-Padé [20] are in good agreement with the results obtained via HDM. More precisely, the approximated solutions are exactly the same. However, in the technique used in [20], one needs first to apply the Laplace transform on the system, following by the ADM, and finally take the inverse Laplace transform to obtain the approximated solutions, which is much time consuming and sometimes can lead to a very difficult situation, for example, if the inverse Laplace transform cannot be obtained.

## 5. Conclusions

In this paper, homotopy decomposition method has been developed for finding approximate solutions of HIV infection model of CD4^+^T which is a class of nonlinear ordinary differential equation systems. We have demonstrated the accuracy and efficiency of the present technique with an example and comparison of the approximate solution obtained via the technique with those obtained with other methods. Comparing the methodology HDM to homotopy perturbation method (HPM), Adomian decomposition method (ADM), variational iteration method (VIM), and homotopy analysis method (HAM) has advantages. Disparate the ADM, the HDM is free from the need of Adomian polynomials. In this method, we do not need the Lagrange multiplier, correction functional, stationary conditions, or calculation of heavy integrals; the solution obtained is noise free, which eliminate the complications that exist in the VIM [23]. In contract to HPM, we do not need to continuously deform a difficult problem to another that is easier to solve. In contrast to LADM, the technique does not need any change of space, which sometime can be very difficult situation to handle, for example, in case the inverse Laplace transform cannot be found. We can easily conclude that the homotopy decomposition method is an efficient tool to solve approximate solution of nonlinear system partial differential equations.

## Figures and Tables

**Table 1:** Numerical comparison of T(t) for *N* = 8.

t	HDM	LADM-Padé [20]	Runge-Kutta	MVIM [21]	VIM [21]	BCM [22]
0	0.1	0.1	0.1	0.1	0.1	0.1
0.2	0.2088072731	0.2088072731	0.2088080833	0.2088080868	0.2088073214	0.2038616561
0.4	0.4061052625	0.4061052625	0.4062405393	0.4062407949	0.4061346587	0.3803309335
0.6	0.7611467713	0.7611467713	0.7644238890	0.7644287245	0.7624530350	0.6954623767
0.8	1.3773198590	1.3773198590	1.4140468310	1.4140941730	1.3978805880	1.2759624442
1	2.3291697610	2.3291697610	2.5915948020	0.2088080868	2.5067466690	2.3832277428

**Table 2:** Numerical comparison of I(t) for *N* = 8.

*t*	HDM	LADM-Padé [20]	Runge-Kutta	MVIM [21]	VIM [21]	BCM [22]
0	0	0	0	0.1 × 10^−13^	0	0
0.2	6.03270728 *·* 10^−6^	6.03270728 *·* 10^−6^	6.032702150 *·* 10^−6^	6.032701651 *·* 10^−6^	6.032634366 *·* 10^−6^	6.247872100 *·* 10^−6^
0.4	1.31591617 *·* 10^−5^	1.31591617 *·* 10^−5^	1.315834073 *·* 10^−5^	1.315830167 *·* 10^−5^	1.314878543 *·* 10^−5^	1.293552225 *·* 10^−5^
0.6	2.12683688 *·* 10^−5^	2.12683688 *·* 10^−5^	2.122378506 *·* 10^−5^	2.1223310013 *·* 10^−5^	2.101417193 *·* 10^−5^	2.035267183 *·* 10^−5^
0.8	3.00691867 *·* 10^−5^	3.00691867 *·* 10^−5^	3.017741955 *·* 10^−5^	3.0174509323 *·* 10^−5^	2.795130456 *·* 10^−5^	2.837302120 *·* 10^−5^
1	3.98736542 *·* 10^−5^	3.98736542 *·* 10^−5^	4.003781468 *·* 10^−5^	4.0025404050 *·* 10^−5^	2.431562317 *·* 10^−5^	3.690842367 *·* 10^−5^

**Table 3:** Numerical comparison of V(t) for *N* = 8.

*t*	HDM	LADM-Padé [20]	Runge-Kutta	MVIM [21]	VIM [21]	BCM [22]
0	0.1	0.1	0.1	0.1	0.1	0.1
0.2	0.06187996025	0.06187996025	0.06187984331	0.06187990876	0.06187995314	0.06187991856
0.4	0.03831324883	0.03831324883	0.03829488788	0.03829595768	0.03830820126	0.03829493490
0.6	0.02439174349	0.02439174349	0.02370455014	0.02371029480	0.02392029257	0.02370431860
0.8	0.009967218934	0.009967218934	0.01468036377	0.01470041902	0.01621704553	0.01467956982
1	0.003305076447	0.003305076447	0.009100845043	0.009157238735	0.01608418711	0.02370431861
